# Location matters: spatial dynamics of tumor-infiltrating T cell subsets is prognostic in colon cancer

**DOI:** 10.3389/fimmu.2024.1293618

**Published:** 2024-02-05

**Authors:** Hehuan Zhu, Jessica Roelands, Eiman I. Ahmed, Imke Stouten, Rachel Hoorntje, Ronald L. P. van Vlierberghe, Marieke E. Ijsselsteijn, Xin Lei, Noel F. C. C. de Miranda, Rob A. E. M. Tollenaar, Alexander L. Vahrmeijer, Davide Bedognetti, Wouter R. L. Hendrickx, Peter J. K. Kuppen

**Affiliations:** ^1^ Department of Surgery, Leiden University Medical Center, Leiden, Netherlands; ^2^ Department of Pathology, Leiden University Medical Center, Leiden, Netherlands; ^3^ Translational Medicine Division, Research Branch, Sidra Medicine, Doha, Qatar; ^4^ Department of Biomedical Science, College of Health Sciences, Qatar University, Doha, Qatar; ^5^ College of Health and Life Sciences, Hamad Bin Khalifa University, Doha, Qatar; ^6^ Department of Immunology and Oncode Institute, Leiden University Medical Center, Leiden, Netherlands; ^7^ Kite, A Gilead Company, Santa Monica, CA, United States; ^8^ Tumor Biology and Immunology Lab, Research Branch, Sidra Medicine, Doha, Qatar

**Keywords:** colon cancer, multiplex immunofluorescence, T cell, spatial analysis, tumor microenvironment, immunologic constant of rejection

## Abstract

**Background:**

Colon cancer is a heterogeneous disease and consists of various molecular subtypes. Despite advances in high-throughput expression profiling, limitations remain in predicting clinical outcome and assigning specific treatment to individual cases. Tumor-immune interactions play a critical role, with tumors that activate the immune system having better outcome for the patient. The localization of T cells within tumor epithelium, to enable direct contact, is essential for antitumor function, but bulk DNA/RNA sequencing data lacks spatial distribution information. In this study, we provide spatial T cell tumor distribution and connect these data with previously determined genomic data in the AC-ICAM colon cancer patient cohort.

**Methods:**

Colon cancer patients (n=90) with transcriptome data available were selected. We used a custom multiplex immunofluorescence assay on colon tumor tissue sections for quantifying T cell subsets spatial distribution in the tumor microenvironment, in terms of cell number, location, mutual distance, and distance to tumor cells. Statistical analyses included the previously determined Immunologic Constant of Rejection (ICR) transcriptome correlation and patient survival, revealing potential prognostic value in T cell spatial distribution.

**Results:**

T cell phenotypes were characterized and CD3^+^CD8^-^FoxP3^-^ T cells were found to be the predominant tumor-infiltrating subtype while CD3^+^FoxP3^+^ T cells and CD3^+^CD8^+^ T cells showed similar densities. Spatial distribution analysis elucidated that proliferative T cells, characterized by Ki67 expression, and Granzyme B-expressing T cells were predominantly located within the tumor epithelium. We demonstrated an increase in immune cell density and a decrease in the distance of CD3^+^CD8^+^ T cells to the nearest tumor cell, in the immune active, ICR High, immune subtypes. Higher densities of stromal CD3^+^FoxP3^+^ T cells showed enhanced survival outcomes, and patients exhibited superior clinical benefits when greater spatial distances were observed between CD3^+^CD8^-^FoxP3^-^ or CD3^+^CD8^+^ T cells and CD3^+^FoxP3^+^ T cells.

**Conclusion:**

Our study’s in-depth analysis of the spatial distribution and densities of major T cell subtypes within the tumor microenvironment has provided valuable information that paves the way for further research into the intricate relationships between immune cells and colon cancer development.

## Introduction

Colon cancer ranks third in global cancer diagnoses and is the second primary cause of cancer-related deaths worldwide (1). In recent years, advances have been made in the management of colon cancer patients, including the introduction of innovative (neo)-adjuvant treatment strategies, among which cancer immunotherapy and surgical techniques (2, 3). These developments have resulted in improved survival rates for colon cancer patients (4), but there is still much room for further improvements. In this context, the identification of patients at high risk of disease progression who may benefit most from novel adjuvant treatment strategies has become increasingly important.

Currently, the prognostic assessment in colon cancer is mainly driven by pathological stage (most commonly based on The American Joint Committee on Cancer (AJCC) staging system (5)). Molecular subtypes such as the consensus molecular subtypes (CMS) also have been shown to associate with survival (6). While cancer stage is the most important factor in the decision of adjuvant chemotherapy, its limitations in making accurate predictions are evident from the wide variation in clinical outcomes observed among patients within the same TNM stage (7–9).

Since the 2000s, significant progress in high-throughput DNA and RNA expression profiling technologies (10, 11), has facilitated addressing the molecular heterogeneity of cancers more effectively. In parallel, recent data suggests that tumor-immune interactions play a pivotal role in the development and progression of colon cancer (12–16), making immune-related biomarkers an area of significant research focus. Almost a decade ago, a comprehensive immune gene expression signature, known as the immunologic constant of rejection (ICR), has been proposed to capture the continuum of cancer immune surveillance (17). This signature was subsequently refined and condensed into a fixed 20-gene panel (Th-1 signaling interferon related *IFNG*, *TBX21*, *CD8A/B*, *IL12B*, *STAT1*, and *IRF1*; CXCR3/CCR5 chemoattraction related *CXCL9*, *CXCL10* and *CCL5*; cytotoxic functions related *GNLY*, *PRF*, *GZMA*, *GZMB*, and *GZMH*; immune regulatory related *IDO1*, *CTLA4*, *CD274*, *PDCD1* and *FOXP3*), demonstrating its prognostic relevance across various cancer types, including breast cancer (18), neuroblastoma (19), and soft-tissue sarcoma (20), as well as its association with responsiveness to immunotherapy (18). The ICR signature includes gene modules that reflect the activation of Th1-signaling, Th-1 chemoattraction, cytotoxic functions, and counter-activation of immune regulatory mechanism.

Recently, we performed a multi-omic characterization of primary colon cancer on a cohort of systemic treatment-naive patients. This data repository is referred to as the atlas and compass of immune–cancer–microbiome interactions (AC-ICAM) (21). In this cohort, we validated the prognostic value of the ICR, independently of MSI status and tumor mutational burden. Deep sequencing of the expressed T cell receptors (TCR) indicated that the top genes correlated with TCR clonality are ICR genes, suggesting that the prognostic impact of ICR is attributed to its ability to detect the presence of clonally expanded T cells (21). As bulk gene expression data does not provide information on the specific localization of T cells in the tumor microenvironment (TME), we aimed to further characterize the immune contexture by assessing the localization of T cell subsets within the TME. It is important to assess the location of T cell subsets, as the antitumor function of T cells can be linked to their location, for instance, only T cells within the tumor epithelium can kill tumor cells via direct cell-cell contact (22–24).

Here, we employed multiplex immunofluorescence to quantify and locate T cell subsets within primary tumors and investigated its relation to genomic classifications, clinicopathological parameters and clinical outcomes.

## Materials and methods

### Patient cohort

A total of 90 colon cancer patients were selected from the AC-ICAM (21). All patients underwent surgical removal of the primary tumor at Leiden University Medical Center between 2001 and 2015. This cohort is representative of the larger cohort, with a similar distribution of age, sex, anatomy location, adjuvant treatment history, AJCC stage, MSI status, CMS and ICR classification. The median follow-up time was 4.1 years. The AC-ICAM study excluded individuals who received prior to surgery radiotherapy/chemotherapy and those with primary tumors of non-epithelial origin. Tumor immune phenotype classification was previously performed using unsupervised consensus clustering based on the expression of ICR genes. The clinical and follow-up data were obtained from medical records through retrospective analysis. The collected patient information was de-identified and the tissue samples were handled in accordance with the guidelines outlined in the Code of Conduct for Proper Secondary Use of Human Tissue of the Dutch Federation of Biomedical Scientific Societies, ensuring anonymity. The study was conducted following the principles described in the Helsinki Declaration and was approved by the IRB at LUMC and Sidra Medicine (IRB: 1768087-1, 1602002725 and B19.079).

### Chromogenic singleplex immunohistochemistry

Chromogenic singleplex immunohistochemistry (IHC) was carried out to determine the conditions and the order in which the primary antibodies would be applied in the multiplex protocol. The tissue sections underwent six staining cycles in total. Briefly, 4 μm formalin-fixed paraffin-embedded (FFPE) tissue sections were deparaffinized and rehydrated, and fixed with PBS/1% formaldehyde (Klinipath, Breda, The Netherlands) for 5 minutes at room temperature. To ensure adequate epitope stability following successive rounds of heat-induced antigen retrieval (AR), the singleplex IHC was conducted in the first, intermediate and second to last round of HIER for each of the biomarkers to be multiplexed in the final panel, corresponding to positions 1, 3 and 5 of antibody staining (**Supplementary Table S1**). Results of positions 1, 3 and 5 reflected those of positions 2, 4 and 6, respectively, which permitted rapid IHC optimization. Staining was performed by AR in EnvisionTM FLEX target retrieval solutions, citrate‐based pH 6.0 AR1 (DAKO, Glostrup, Denmark) or EDTA‐based pH 9.0 AR2 (DAKO), using a PT Link module (DAKO).

### Singleplex and multiplex immunofluorescence

In this study, immunofluorescence staining was conducted using the Tyramide Signal Amplification (TSA) based Opal method (Opal Polaris 7-color Manual Detection Kit; Akoya Biosciences; Catalogue No. NEL861001KT). Given that both TSA and DAB oxidation are mediated by peroxidase, the conditions and sequence of primary antibody staining established through DAB detection were directly translatable to fluorescent assays, substituting DAB IHC reagents with Opal reagents (**Supplementary Table S2**). Unlike conventional IHC that employs chromogenic peroxidase substrates, each antibody in this study was associated with a distinct Opal fluorophore. The selection of optimal Opal-antibody combinations was guided by the anticipated co-expression and abundance levels of specific biomarkers in colon tissue samples. Moreover, fluorophores with higher intensity were paired with low-abundance markers to enhance spectral acquisition, and vice versa. Opal fluorophores were used at a 1:100 dilution, in accordance with manufacturer guidelines (25). To evaluate staining performance, a fluorescent singleplex was run for each biomarker and juxtaposed with its chromogenic counterpart. Dropout controls were used to ascertain the absence of signal interference between antibodies. During the phases of singleplex development and multiplex optimization, various parameters such as Opal-antibody pairings, concentrations, and denaturing conditions were meticulously assessed and fine-tuned. This was achieved by monitoring the signal-to-background ratio (signal intensity of positive staining to background > 10:1) and signal balance (signal intensity of all fluorophores < 30 counts) using Akoya’s Inform software (version 2.4.6). The target signal intensity range was set between 15 and 30 counts for each antibody, equivalent to 100–125 nm of fluorescence capture on the Vectra Polaris platform.

In each staining cycle, tissue sections were exposed to one type of primary antibodies targeting CD3, CD8, FOXP3, Granzyme B(GrB), Ki-67, and CK. These were followed by incubation with horseradish peroxidase (HRP)-conjugated secondary antibodies (anti-mouse/rabbit Envision, DAKO). Subsequently, sections were developed using a spectrum of Opal fluorophores (Opal 520, Opal 540, Opal 570, Opal 620, Opal 650, and Opal 690), all dissolved in 1x amplification buffer. Post-visualization, the sections underwent microwave treatment in AR6 or AR9 buffer (DAKO) to remove antibody complexes and facilitate antigen retrieval for subsequent staining cycles. Finally, all sections were counterstained with DAPI (Sigma-Aldrich) and mounted using ProLong Gold Antifade Mountant (Thermo Fisher Scientific, Bleiswijk, The Netherlands).

### Image capture and analysis

The VECTRA 3.0 automated quantitative pathology imaging system (Akoya Biosciences) was used for imaging of the multiplexed-stained slides. The whole tissue sections were scanned at 10x magnification. PhenoChart software (Akoya Biosciences, 1.0.4.) was used to randomly select 4 multispectral imaging fields within tumor regions, defined as area containing at least 30% tumor epithelium area based on anti-cytokeratin staining and DAPI signal, which were then scanned at a higher resolution (20x). Spectrally unmixed images were generated using the inForm software package (v2.6.0; Akoya Biosciences) and further analyzed using pathologist-supervised machine learning algorithms built into the inForm software package. Tissue regions were categorized into epithelium, stroma, and “other” (such as empty space or necrosis) categories. Individual cells were segmented into nuclear, cytoplasmic, and membranous regions. In this cohort, GrB^+^ subsets were identified using a cytoplasmic mean intensity cut-off of 0.25, on a scale of 0 to 1 indicating the range of fluorescence intensity (26). Cells were then phenotyped into the CD3^+^ (T cells), CD3^+^Ki67^+^ (proliferating T cells, pT), CD3^+^CD8^-^FoxP3^-^ (presumably CD4 T cells, helper T cells, Th), CD3^+^CD8^-^FoxP3^-^Ki67^+^ (proliferating Th cells, pTh), CD3^+^FoxP3^+^ (presumably regulatory T cells, Tregs), CD3^+^FoxP3^+^Ki67^+^ (proliferating Tregs, pTreg), CD3^+^CD8^+^ (Cytotoxic T cells, CD8 T), CD3^+^CD8^+^Ki67^+^ (proliferating cytotoxic T cells, pCD8 T), CD3^+^CD8^+^GrB^+^ (active cytotoxic T cells, aCD8 T), and CK^+^ (tumor epithelial cells) categories. The cell phenotype classification implemented in the inForm software package was based on multinomial logistic regression utilizing image features derived from texture analysis and cell segmentation. The phenotprReports add-in provided by Akoya Biosciences was used for the analysis of densities and distance measurements, as well as for creating a quality of unmixing report. For distances between tumor cells to T cells, we restricted this analysis to T cells that were not intraepithelial as distances will be influenced by the amount of cells inside the epithelium.

### Transcriptome and whole exome sequencing data

CMS classification, ICR clusters, and MSI status based on MANTIS (27) were previously determined (21). Data is available in the **Supplementary Source Data File** corresponding to the original publication (21) (Gene expression matrix in Supplementary Source Data 3, CMS classification in Supplementary Source Data 4, ICR clusters in Supplementary Source Data 19, and MSI in Supplementary Source Data 11).

### Gene expression correlation

The correlation between the distinct metrics (densities and distances) and selected genes, including the 20 ICR genes, all chemokine genes from the KEGG pathway (KEGG_CHEMOKINE_SIGNALING_PATHWAY, cytokines and growth factors), and all KEGG cell-cell interaction molecules, were generated using Pearson correlation.

### Statistics

All statistical analyses were performed using the IBM SPSS (version 25; Armonk, NY) and GraphPad Prism software (version 7.0d; GraphPad, San Diego, CA, USA) unless stated otherwise. Statistical analyses of quantification were performed with the two-tailed Mann–Whitney U-test between two groups and the Kruskal–Wallis test among multiple groups as appropriate. For survival analyses, patients were categorized into two groups based on immune cell density and the distance from immune cells to tumor cells or between immune cells; the median density or distance was used as the cut-off value. Kaplan–Meier plots were drawn, and statistical differences were evaluated using the log-rank Mantel–Cox test. Furthermore, the spatial distribution of T cells was correlated with next generation sequencing data using Pearson correlation tests. Univariate and multivariate prognostic analyses were done using Cox regression analysis (Wald test). The variables tested in the univariate analysis included patients’ age and sex, anatomy location, adjuvant treatment, AJCC stage, MSI status, CMS classification, the ICR-based classification, and the classification of the spatial distribution of T cells based on their densities and distances among each other. Multivariate analysis incorporated all variables with a p value inferior to 0.05 in univariate analysis. Cox proportional hazard models were built to investigate whether the spatial distribution improved the prognostic value of T cell immune signatures. A *P*-value of ≤ 0.05 was considered statistically significant.

## Results

### Patient cohort and experimental approach

**Figure 1:**
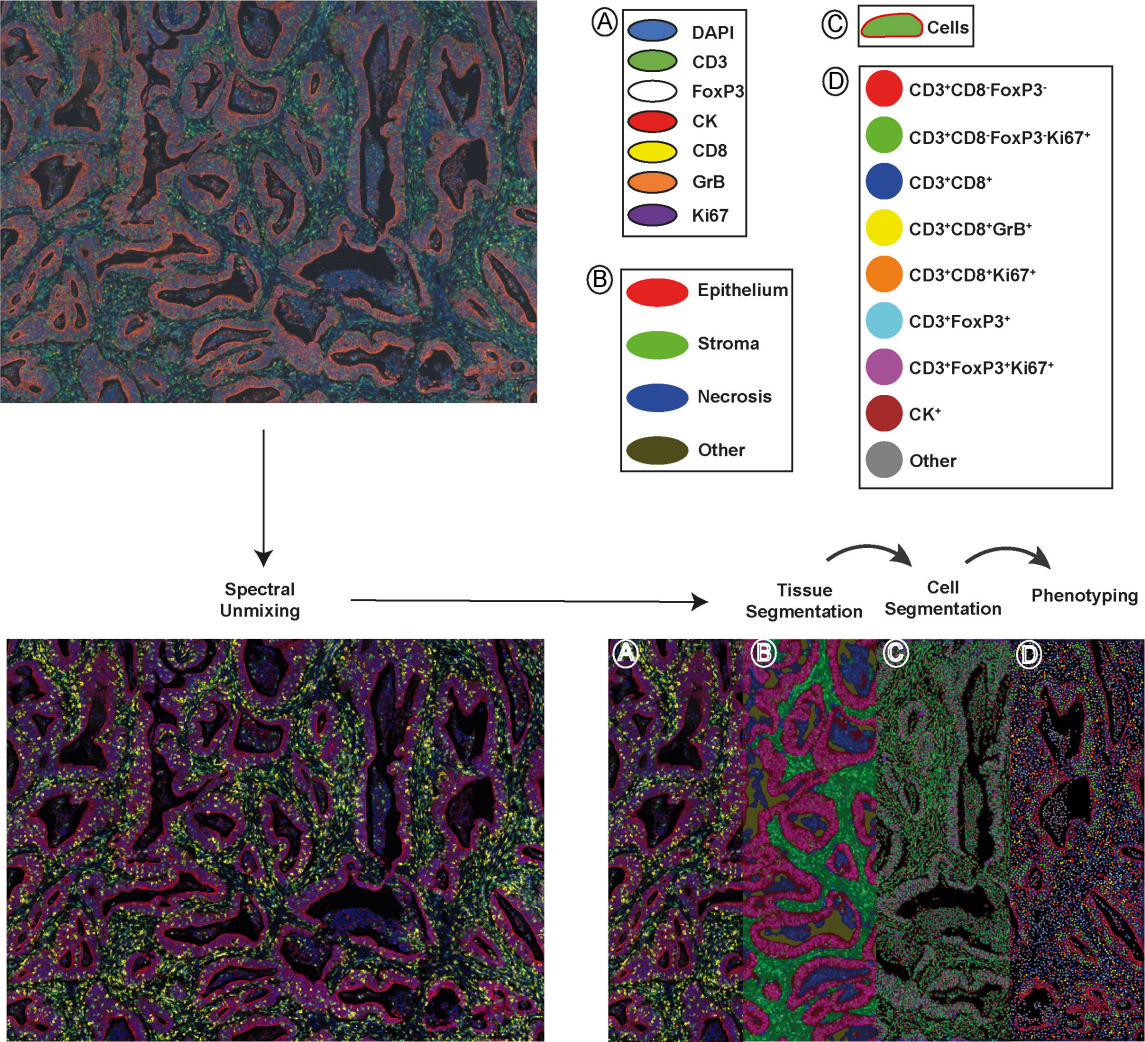
A schematic depiction of the analytical pipeline, encompassing spectral unmixing, tissue segmentation, cell segmentation, and phenotyping, is provided. The top-left panel displays the original image and the bottom-left panel shows the unmixed image featuring all stained markers: DAPI (blue), CD3 (green), FoxP3 (white), CK (red), CD8 (yellow), GrB (orange), and Ki67 (purple). In the bottom-right panel, tissue segmentation and cell segmentation are illustrated in the middle-left and middle-right of the panel, separately. Tumor epithelial regions are shown in red, stromal areas in green, necrotic zones in blue and empty space in dark yellow. The right part of the panel exhibits phenotyped cells from one project, represented as dots with corresponding marker combination colors. Scale bar is 100 µm.

To comprehensively characterize the tumor‐infiltrating T cells in colon tumors, we investigated different immune cell types, their densities, and spatial relationships, as well as matched molecular profiling data in the 90 clinical samples of colon primary tumors by integrating multiplex immunofluorescence (mIF), gene expression signature and clinical outcome analysis. These 90 samples as a subset of AC-ICAM and are preserved in a different block of formalin-fixed paraffin-embedded (FFPE) tissue, as opposed to being frozen. Patient and tumor characteristics are summarized, together with extensive clinicopathological and genomic data in **Supplementary Table S3**. Of note, our mIF subset and the original AC-ICAM cohort (21) did not have a significant difference in distribution of these parameters. We screened potential markers and antibody clones based on our previous published literature (19, 28, 29). Candidate antibody testing was then performed on colon cancer tissue and lymphoid controls via chromogenic immunohistochemistry, and antibody clones with expression patterns corresponding to their biologically expected distribution (at both the tissue level and subcellular level) were selected for further testing via immunofluorescence (**Supplementary Figure S1**). Antibody and fluorophore concentrations and antibody–fluorophore pairing were iteratively optimized, and similar staining patterns between chromogenic immunohistochemistry and multiplex immunofluorescence were confirmed. An example of the analytical pipeline is shown in **Figure 1**. We separately constructed and profiled a spectral fluorophore and autofluorescence library to enable optimal multispectral unmixing (**Figure 1**, **Supplementary Figure S1**, **Supplementary Table S3**). Each cell was annotated with spatial coordinates, allowing cells not only to be categorized but also quantified into either the epithelium or stroma compartment within the tumor tissue. Additionally, spatial proximity between cells could be quantified (**Supplementary Figure S2**).

### Immune cell phenotypes characterized in colon cancer

**Figure 2:**
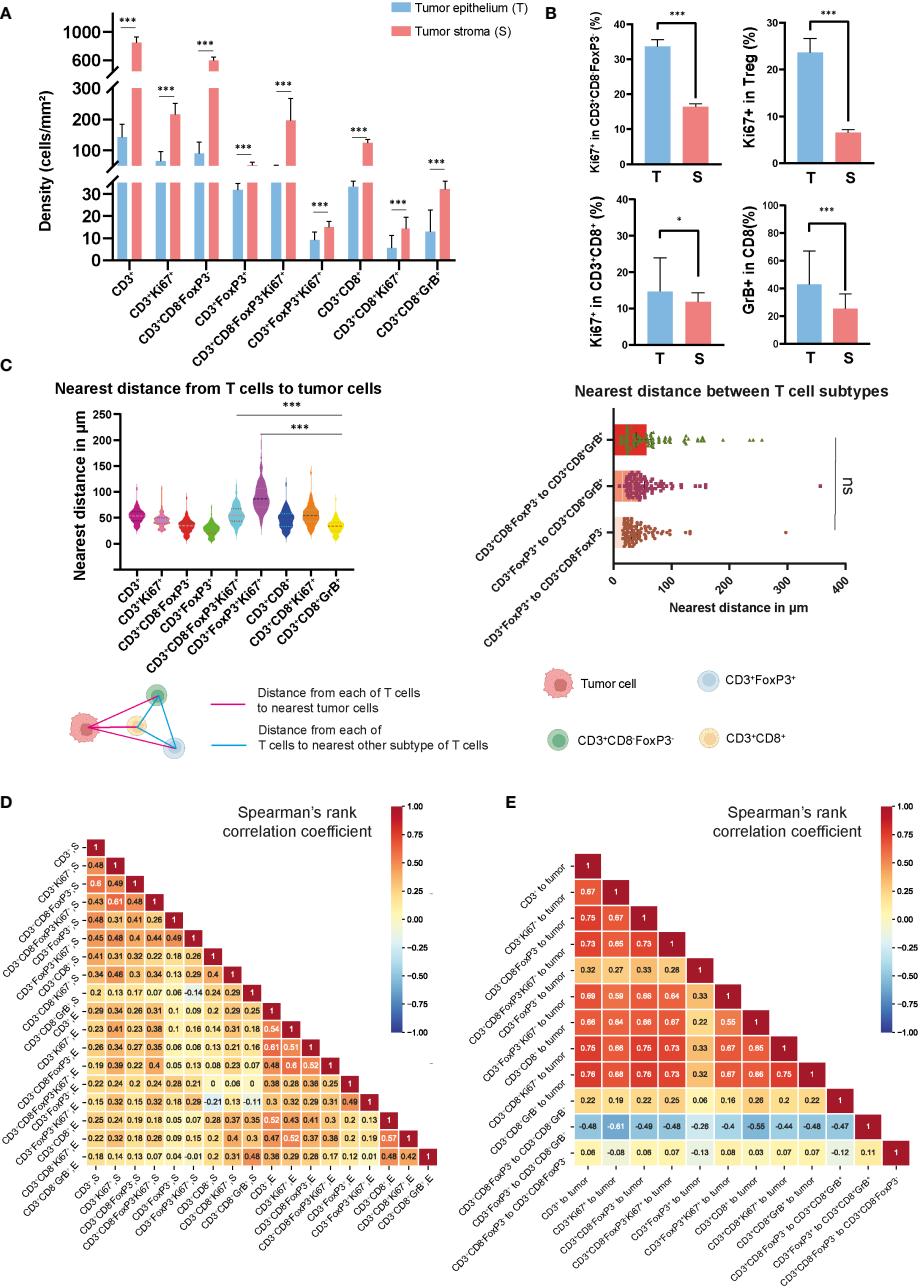
Densities and spatial distribution of T cell subsets in colon tumors. **(A)** Overview of the densities of T cell subsets in colon tumor epithelium and stroma, separately. **(B)** Comparison of the percentage of Ki67+ and GrB+ in different T cell subsets in epithelial and stromal compartments. **(C)** The top-left panel showed the nearest distance from different T cell subsets to tumor cells. The top-right panel showed the nearest distance between different T cell subsets. On the bottom side, a schematic diagram illustrated the spatial distances between various T cell populations or from T cell subsets to tumor cells **(D)** Correlation matrix of Spearman correlation coefficients between the densities of intraepithelial and stromal T cells. **(E)** Correlation matrix of Spearman correlation coefficients between the distances from T cells to tumor cells or between T cell subtypes. *** represent *p* value< 0.001, * represent *p* value< 0.05, ns represent *p* value> 0.05.

In studying the TME from colon cancer, it was possible to identify different tumor-associated T cell populations using the expression of the cell-type specific markers CD3, CD8, FoxP3, and their co-expression with the other markers in the panel, as shown in **Supplementary Figure S1** and **Figure 1B**. Overall, the dominant T-cell subset observed were CD3^+^CD8^-^FoxP3^-^ T cells, with a median of 89.6 cells/mm^2^ in the epithelium and 595.2 cells/mm^2^ in the stroma, compared to other T cell subtypes such as CD3^+^FoxP3^+^ and CD3^+^CD8^+^ T cells, as shown in **Figure 2A** and **Supplementary Table S3**. CD3^+^FoxP3^+^ T cells occurred in similar densities as CD3^+^CD8^+^ T cells in both compartments. In the tumor epithelium 14.8% of CD3^+^CD8^+^ T cells expressed the proliferating marker Ki67 compared to 33.7% of CD3^+^CD8^-^FoxP3^-^ T cells (**Figure 2A** and **Supplementary Table S4**). Of note, in this cohort, we observed that the densities of all the T cell subtypes were significantly higher in the tumor stroma compared with the epithelial compartment. Interestingly, the percentage of Ki67-expressing cells in T cells, including CD3^+^CD8^-^FoxP3^-^, CD3^+^FoxP3^+^, and CD3^+^CD8^+^ T cells, was significantly higher when they infiltrated the epithelium compared to the stroma (**Figure 2B**). Moreover, in the epithelium, the proportion of CD3^+^CD8^+^GrB^+^ T cells was notably greater than that observed in the stromal compartment.

In order to map the spatial organization of the lymphocyte infiltration and capture the interaction between cells, we constructed a matrix where each entry is the Euclidean distance from a pair of cells and assessed their distribution using the X and Y coordinates of each cell within the TME. From this matrix, we were able to identify the median distances from each immune cell to the closest tumor cell and the distances between each immune cell subtype described above (**Figure 2C** and **Supplementary Figure S2**). Using the median value of the nearest distance, which refers to the shortest spatial distance between two individual cells, we were able to generate a heat map (**Supplementary Figure S3**) including the different T cell subtypes obtained with the image analysis, and we identified the distance of 53.2 μm as the overall nearest radius distance from T cells and tumor cells (**Supplementary Table S5**). In our cohort of colon tumors, the T cell subtype which had the closest distance to tumor cells were the CD3^+^CD8^-^FoxP3^-^Ki67^+^ T cells with a median distance of 27.9 μm (**Figure 2C**). We also observed that CD3^+^CD8^+^GrB^+^ T cells were relatively close to tumor cells compared to CD3^+^FoxP3^+^ and CD3^+^FoxP3^+^Ki67^+^ T cells, with median distances of 33.9, 54.6, and 86.6 μm, respectively. Correlative analysis demonstrated that the majority of lymphocyte subtypes appeared concurrently, suggesting a coordinated infiltration of immune cells within the TME (**Figure 2D**). Notably, a generally high T cell infiltration in epithelium was accompanied by the abundance of CD3^+^CD8^-^FoxP3^-^ and CD3^+^CD8^+^ T cells, whereas only CD3^+^CD8^-^FoxP3^-^ T cells were strongly associated with CD3^+^ T cells in the stromal area. Furthermore, we observed an unexpected connection between the distance from CD3^+^FoxP3^+^ to CD3^+^CD8^+^GrB^+^ T cell and the distances among other immune cells or immune cells to tumor cells (**Figure 2E**). Overall, the densities of different T cell types showed low to moderate correlation, while the distances among them exhibited moderate to high correlation.

### Association between gene expression of chemokines, cell-cell interaction molecules and ICR genes

**Figure 3:**
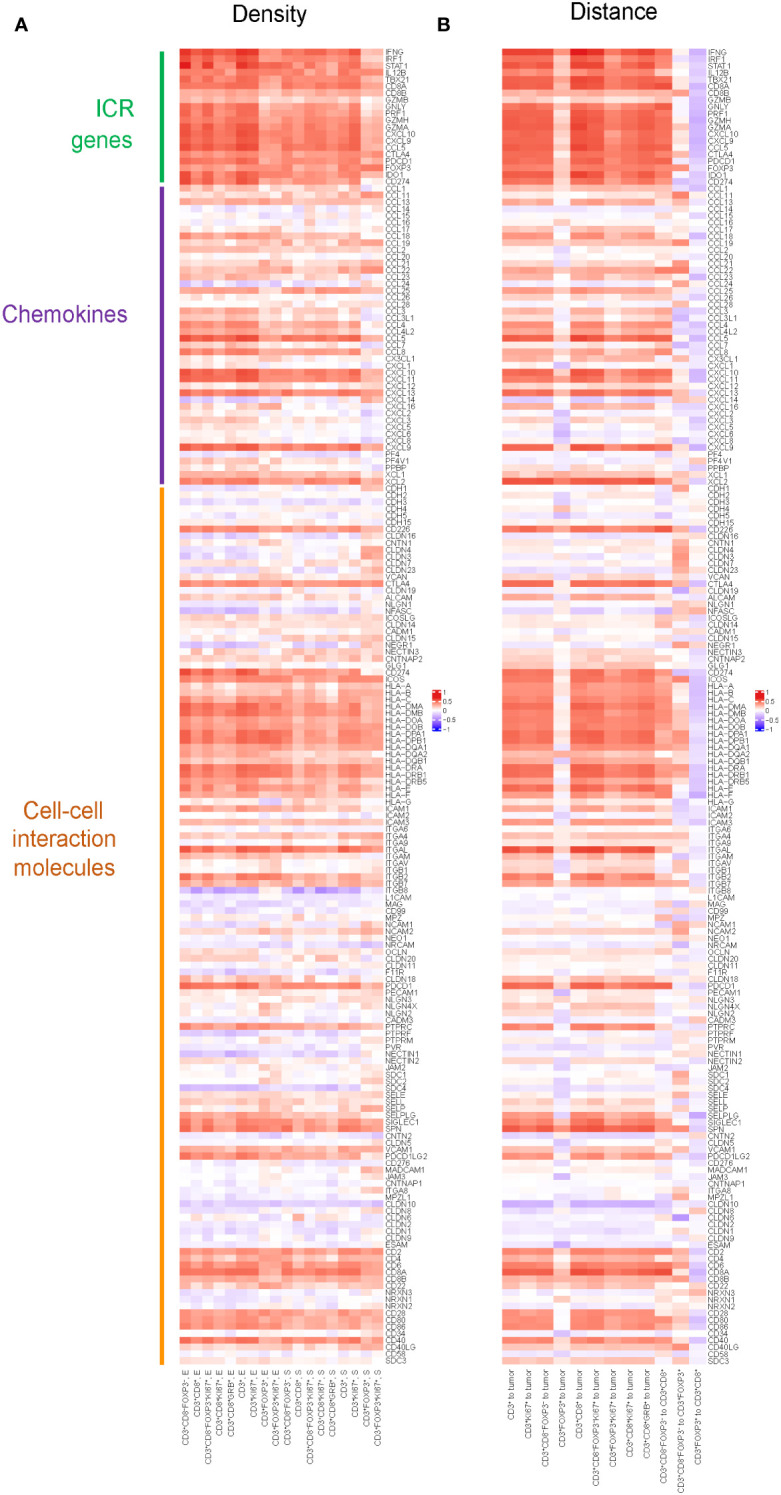
Pearson correlation between spatial distribution of T cell subtypes and gene expression of selected genes including chemokines, cell-cell interaction molecules and ICR genes. **(A)** Pearson correlation between densities of T cell subtypes and gene expression of selected genes including chemokines, cell-cell interaction molecules and ICR genes. Positive correlation in densities implies higher gene expression is associated with higher density. The color in the heatmap defines the correlation, red means a strong positive correlation, blue an inverse correlation, and white meaning no correlation. **(B)** Pearson correlation between distances of T cells to tumor cells, as well as the distances among different T cell subtypes, and the gene expression of selected genes including chemokines, cell-cell interaction molecules and ICR genes. Positive correlation in distance implies higher gene expression is associated with shorter distance.

To explore potential mechanisms of immune cell recruitment and retention to the tumor, we assessed the relation between T cell densities and immune-related gene expression, including ICR, KEGG chemokines and KEGG cell-cell interaction molecules. In ICR gene clusters (**Figure 3A**), the results showed strong correlations between the density of T cell subtypes and the expression of genes, such as *IFNG*, *IRF1*, and *STAT1*. These genes play pivotal roles in the tumor immune response, particularly in T cell activation, cytokine production, and anti-tumor immune response, suggesting that the ICR captures a higher T cell infiltrated TME. Additionally, we observed that the density of T cell subsets highly correlated with the gene expression of specific chemokines, like *CXCL10* and *CXCL11*, which may reflect the role of chemokines in modulating immune cell migration into the TME. A noteworthy aspect of the results within the cell-cell interaction molecules is the correlation between the density of all T cell subtypes and the expression of genes in the Human Leukocyte Antigen (HLA) family, which suggested a possible mechanism where increased T cell presence may either be a consequence of or a contributor to the heightened expression of these HLA molecules.

Similarly, we assessed the correlation between the distances of various T cell subtypes from tumor cells and their intra-subtype distances, and the expression of genes within the ICR signature, KEGG chemokines, and KEGG cell-cell interaction molecules(**Figure 3B**). We found nearly all genes in the ICR cluster, with the notable exception of *GZMB*, exhibited a significant correlation with the distances between various T cell subtypes and tumor cells: the closer the T cells are to the tumor, the more pronounced the expression of these key immunoregulatory genes appears to be. Furthermore, we observed that as the distance between CD3^+^CD8^-^FoxP3^-^ T cells and CD3^+^CD8^+^ T cells increased, there was a corresponding elevation in the expression levels of ICR genes and most genes in chemokines and cell-cell interaction molecules.

### ICR classification and clinicopathological and spatial distribution of T cell subsets correlations

**Figure 4:**
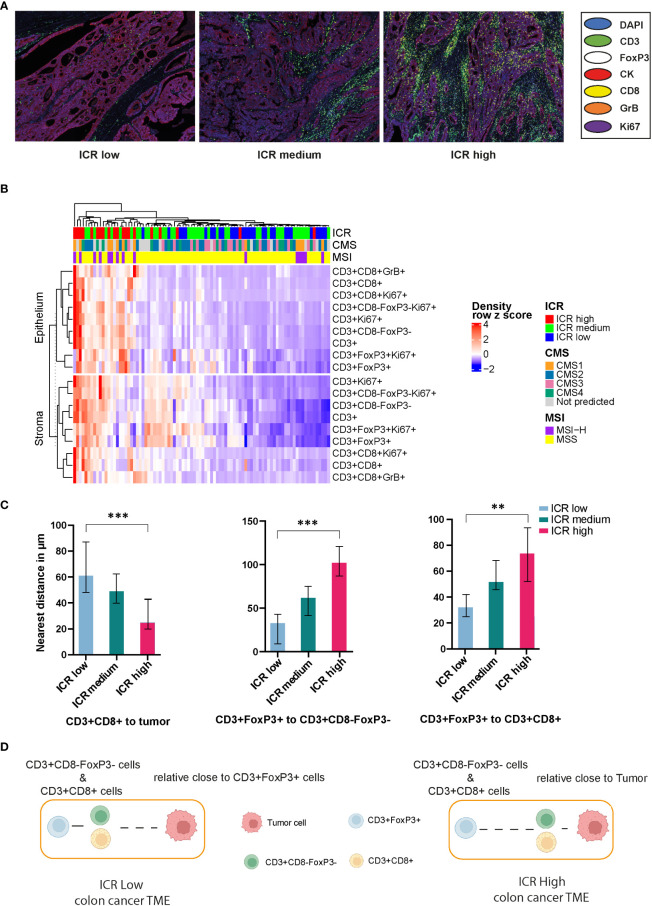
Correlation of Immunologic Constant of Rejection (ICR) classification and spatial distribution of T cell subsets in colon cancer. **(A)** Representative mIF images of different ICR immune subtypes. **(B)** Heatmap of correlations among ICR classification, CMS classification and MSI status with T cell subtypes. **(C)** Correlation of ICR classification and the distance from CD3^+^CD8^+^ T cells to tumor cells, the distance between CD3^+^FoxP3^+^ to CD3^+^CD8^-^FoxP3^-^ T cells and CD3^+^FoxP3^+^ to CD3^+^CD8^+^ T cells. **(D)** A proposed model of tumor microenvironment (TME) characteristics for colon cancer patients. In ICR low patients, the TME exhibits persistent immune regulation, with CD3^+^CD8^-^FoxP3^-^ and CD3^+^CD8^+^ T cells in closer proximity to CD3^+^FoxP3^+^ T cells. In contrast, ICR high patients exhibit an enhanced functional immune state, facilitating closer proximity of CD3^+^CD8^-^FoxP3^-^ and CD3^+^CD8^+^ T cells to tumor cells. The p-values of comparison between the three immune subtypes (one-way ANOVA test) are shown on the top (NS represent not significant; ** represent *p* value <0.01; *** represent *p* value <0,001).

Representative mIF images of the three different ICR immune subtypes are shown in **Figure 4A**. This classification demonstrated a heightened enrichment of the MSI-H signature progressing from ICR Low to ICR High (**Supplementary Table S6**). In this subset of AC-ICAM, differences were observed between the three ICR immune subtypes regarding the patients’ age (p= 0.040), sex (p= 0.019), and MSI status (p= 0.029): ICR Low class was associated with younger age, male, and Micro Satellite Stable. No difference was found regarding the anatomical location of the tumor, the history of adjuvant treatment, the pathological tumor stage, or the CMS classification.

We then explored correlations between ICR and densities of T-cell subsets and their spatial distribution, such as the distance of T-cell subsets from tumor cells and the distance between different T-cell subsets (**Supplementary Figure S3** and **Supplementary Table S7**). We observed that the densities of all T cell subsets were positively associated with increasing ICR (**Figure 4B**), while CD3^+^CD8^+^ T cells were in closer proximity to tumor cells (**Figure 4C**). Notably, we observed increased separation between CD3^+^CD8^-^FoxP3^-^ or CD3^+^CD8^+^ T cells and the closest CD3^+^FoxP3^+^ with increased ICR score (**Figure 4C**, right 2 panels), indicating that the higher ICR immune subtypes represent a more robust antitumor immune response since CD3^+^FoxP3^+^ T cells are known for their immune regulation function. Summarized, we demonstrate here that ICR High samples were indeed characterized by a high density of lymphocytes such as CD3^+^CD8^-^FoxP3^-^ and CD3^+^CD8^+^ T cells, with these T cells being relatively proximal to cancer cells compared to CD3^+^FoxP3^+^ T cells (**Figures 4A, D**).

### Spatial distribution of T cells is associated with survival

**Figure 5:**
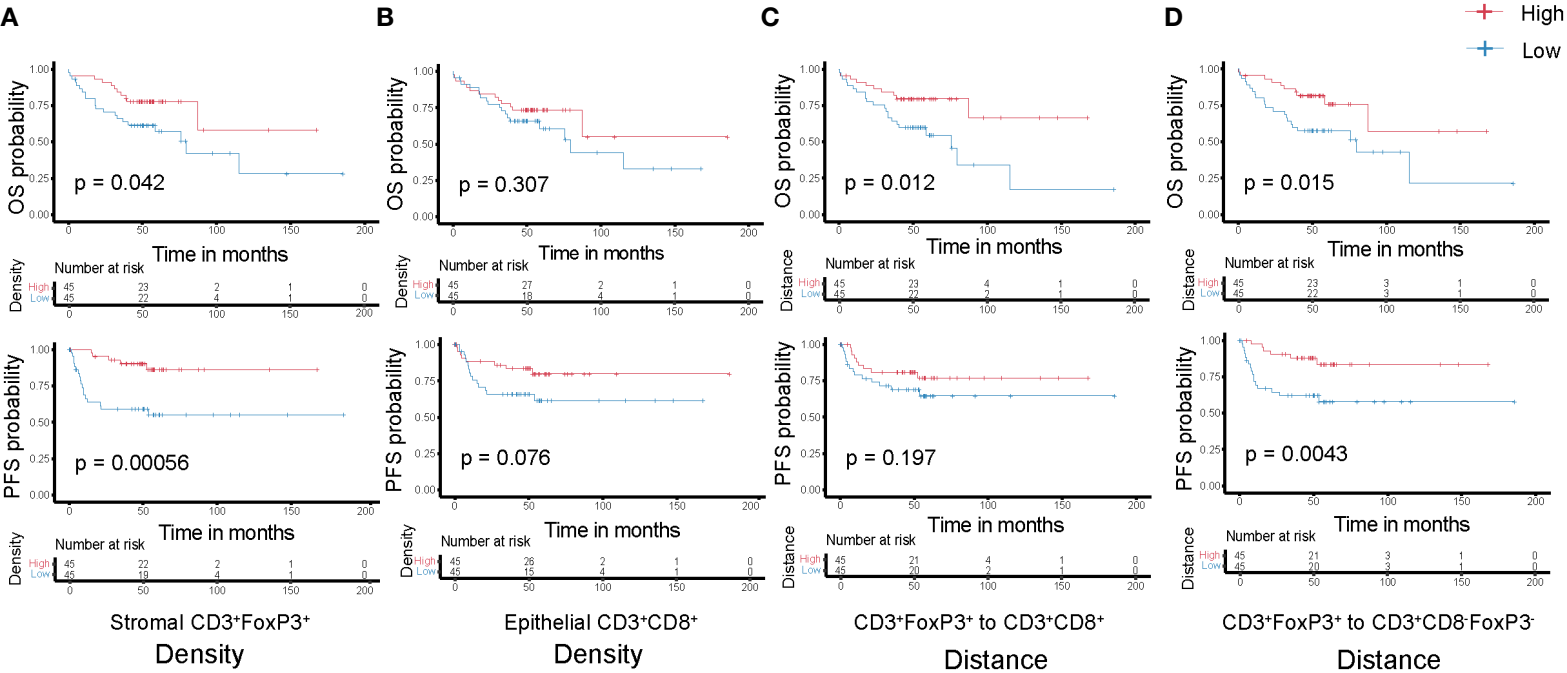
Kaplan-Meier curves of overall (top-panels) and progression-free (bottom-panels) survival according to the densities of T cell subtypes **(A)** Stromal CD3^+^FoxP3^+^ T cells; **(B)** Epithelial CD3^+^CD8^+^ T cells and the distances among different T cell subtypes **(C)** CD3^+^FoxP3^+^ to CD3^+^CD8^+^ T cells; **(D)** CD3^+^FoxP3^+^ to CD3^+^CD8^-^FoxP3^-^ T cells. Densities and distances above the median are designated as ‘high’, while those below the median are classified as ‘low’.

Subsequently, we analyzed the relationship between T cell density and survival of colon cancer patients. Patients were classified into two groups, high or low density, based on the median value of densities for each T cell subtype in the epithelial or stromal compartment. The value of this spatially resolved analysis was highlighted by other researchers, which reported that the associations were more significant for intraepithelial and stromal compartments compared to the overall tissue area (26, 30). Tregs are generally known to accompany an antitumor immune response as a counter regulatory factor, in our cohort we noticed that the high density of CD3^+^FoxP3^+^ T cells in the tumor stromal compartment was associated with improved OS and PFS (**Figure 5A**); while in the epithelial compartment, the association was not statistical significant (data not shown). Simultaneously, higher epithelial CD3^+^CD8^-^FoxP3^-^, CD3^+^CD8^+^, and CD3^+^CD8^+^GrB^+^ T cells densities exhibited this trend, albeit not reaching statistical significance (**Figure 5B**; **Supplementary Figures S4A, B**), suggesting that an antitumor immune response can contribute to better clinical outcomes.

Each T cell subtype has unique functional properties, and their spatial distribution can affect their ability to exert antitumor effects. Thus, studying the distance between T cells and the tumor cells, or between different T cell subtypes, will provide insight into these immune evasion mechanisms and how they affect patient survival. We classified patients into two groups, high or low distance to tumor cells, based on the median of the distance for the two closest T cell subtypes. Unsurprisingly, low distance from CD3^+^CD8^+^ T cells to tumor cells correlated with improved OS and PFS (**Supplementary Figure S4C**), although not significantly, which may be due to the sample size and highly heterogeneous TME. In addition, we postulate that greater distance between CD3^+^FoxP3^+^ and other effector T cells, such as CD3^+^CD8^-^FoxP3^-^ or CD3^+^CD8^+^ T cells, may diminish the immune regulation effects exerted by these CD3^+^FoxP3^+^ T cells. This could allow the effector T cells to sustain their antitumor activity, potentially contributing to improved patient survival (**Figures 5C, D**). CD3^+^CD8^-^FoxP3^-^ and CD3^+^CD8^+^ T cells have distinct but complementary roles in orchestrating immune responses against cancer cells, and their spatial distribution and interaction can influence the overall effectiveness of the immune response (22, 31). However, there was no significance regarding patient survival between relatively low or high distance from CD3^+^CD8^-^FoxP3^-^ to CD3^+^CD8^+^ T cells in our cohort (**Supplementary Figure S4D**).

The findings confirm that many T cell subsets possess a positive influence on clinical outcome of colon cancer patients, with this impact extending beyond merely cytotoxic T-lymphocytes. In univariate analysis of this cohort, we observed that the distance between CD3^+^FoxP3^+^ and CD3^+^CD8^-^FoxP3^-^ T cells was negatively associated with patients’ PFS: patients with a higher distance from CD3^+^FoxP3^+^ to CD3^+^CD8^-^FoxP3^-^ T cells showed longer PFS (**Figure 5D**), representing a 71.9% decreased risk of event (HR= 0.281, 95% CI 0.111 to 0.713; p= 0.004, Wald test; **Table 1**). The other variables associated with shorter PFS included adjuvant treatment (p= 0.044) and AJCC stage (p= 0.002). In multivariate analysis (**Table 1**), the distance between CD3^+^FoxP3^+^ and CD3^+^CD8^-^FoxP3^-^ T cells remained associated with PFS (p= 0.004, Wald test), as well as AJCC stage, suggesting independent prognostic value.

**Table 1:** Univariate and multivariate prognostic analyses for progression-free survival (PFS).

Characteristics	Univariate		Multivariate	
	HR (95% CI)	*P* value	HR (95% CI)	*P* value*
Age (years)	0.976 (0.944-1.008)	0.412		
Sex				
Male vs Female	1.092 (0.478-2.497)	0.834		
Anatomy location				
Right vs left	1.811 (0.794-4.131)	0.158		
Adjuvant treatment				
Yes vs No	2.318 (1.022-5.254)	**0.044**	1.016 (0.351-2.942)	0.976
AJCC staging				
III/IV vs I/II	4.213 (1.732-10.251)	**0.002**	4.802 (1.562-14.764)	**0.006**
MSI status				
MSI-H vs MSS	0.834 (0.247-2.814)	0.834		
CMS classification				
CMS2 vs CMS1	0.736 (0.135-4.019)	0.723		
CMS3 vs CMS1	0.453 (0.064-3.219)	0.429		
CMS4 vs CMS1	2.140 (0.442-10.359)	0.345		
Mixed vs CMS1	2.371 (0.503-11.178)	0.275		
ICR classification				
ICR Medium/High vs ICR Low	0.901 (0.381-2.130)	0.812		
Densities of CD3^+^CD8^-^FoxP3^-^ T cells				
High vs Low	1.645 (0.712-3.8030	0.244		
Densities of CD3^+^FoxP3^+^ T cells				
High vs Low	2.184 (0.925-5.156)	0.075		
Densities of CD3^+^CD8^+^ T cells				
High vs Low	2.138 (0.904-5.052)	0.083		
Distance from CD3^+^CD8^-^FoxP3^-^ T cells to tumor				
High vs Low	2.103 (0.890-4.968)	0.090		
Distance from CD3^+^CD8^+^ T cells to tumor				
High vs Low	2.001 (0.847-4.729)	0.114		
Distance between CD3^+^CD8^-^FoxP3^-^ and CD3^+^FoxP3^+^ T cells				
High vs Low	0.281 (0.111-0.713)	**0.004**	0.238 (0.091-0.626)	**0.004**
Distance between CD3^+^CD8^+^ and CD3^+^FoxP3^+^ T cells				
High vs Low	0.576 (0.249-1.331)	0.197		

*, Wald test. AJCC, The American Joint Committee on Cancer; MSI, Microsatellite Instability; CMS, Consensus Molecular Subtypes; ICR, Immunologic Constant of Rejection. Bold values denote statistical significance at the p < 0.05 level.

## Discussion

It is crucial to underline that our study was not conceived as a biomarker investigation. Given the multitude of markers and combinations tested, there is a substantial risk of overfitting, notwithstanding our rigorous adjustment for multiple testing. Given the absence of an independent validation cohort, the analyses should be viewed as descriptive. Furthermore, while we performed phenotypic characterization of infiltrating T cells, their functional characteristics were not assessed. Nonetheless, we believe that the study yielded numerous intriguing observations.

Leveraging a unique pipeline combining multiplex immunofluorescence staining, spectral scanning, and advanced image analysis, we managed to quantify and pinpoint the location of major immune cell classes and their subtypes within the *in situ* environment of diagnostic colon cancer tissue. The separate analysis of immune cell subsets based on marker expression facilitates a more nuanced understanding of cellular immune responses, while correlation analysis offers a more comprehensive view of intercellular relationships. This analysis not only included traditionally analyzed T cell populations but also their subtypes, as well as their distinct spatial distribution and vicinity of immune cell subsets, indicating potential mechanistic interactions. Furthermore, we identified correlations between ICR immune subtypes and the densities and spatial distribution of T cell subsets. This dataset, as an addition to the AC-ICAM cohort, will serve as a valuable supplement to existing bulk RNA sequencing datasets that currently lack the provision of topographical contexture.

The prognostic significance of T cells in the colon cancer microenvironment has been extensively studied for the last two decades, with numerous reports to date. Most of these studies, using conventional single-color immunohistochemistry or bulk transcriptomics and deconvolution techniques, have suggested that higher densities of Th, Treg, or CD8 T cells are associated with a favorable outcome (32–36). We leveraged recent technical advances in multiplexed immunofluorescence to build a novel assay enabling *in situ* characterization of detailed phenotypes for T lymphocytes in colon cancer. In this study, we observed the dominant T-cell subset was CD3^+^CD8^-^FoxP3^-^ T cells, which have been reported to play a critical role in the TME by initiating and regulating immune responses, such as by providing help to CD3^+^CD8^+^ T cells and B cells (37, 38). Moreover, the balance between effector and regulatory T cell populations was evident, as CD3^+^FoxP3^+^ T cells were found in approximately equal numbers to CD3^+^CD8^+^ T cells in both compartments. Another notable observation was the significantly higher percentage of Ki67-expressing T cells in the tumor epithelium compared to the stroma across all T cell subtypes. This increased proliferation rate could be attributed to several factors, including the presence of antigen-presenting cells and local cytokine production, which may stimulate T cell activation and proliferation (39, 40).

Previous studies predominantly indicate an association between the increased number of Th and CD8 T cells and extended survival in colon cancer (32, 34, 37, 41), whereas a higher count of Treg cells correlated with shorter survival (33, 42). Building on the evolving understanding of the immune landscape in colon cancer, the current literature suggests a nuanced role of Treg cells, marked by their heterogeneity and contrasting impacts on patient outcomes. Now many evidence shown that Tregs are associated with an improved survival as the expression of immune-regulatory markers (e.g., FOXP3, CTLA4, and PD-1) reflects the presence of counter-regulatory mechanisms that follow the intra-tumoral infiltration of activated lymphocytes (43–46). Some Treg subsets, particularly those with high FOXP3 expression, have been linked to an anti-tumorigenic response, possibly due to their role in maintaining immune homeostasis and preventing overactive inflammatory responses that can contribute to tumor growth and metastasis (47). This protective role of Tregs can be particularly prominent in the unique microenvironment of colon cancer, where chronic inflammation is a key driver of carcinogenesis. Given the coordinated nature of immune cell infiltration, where most immune cell types infiltrate in unison and their numbers strongly correlate, discerning the primary cellular actors is challenging. In line with these findings, our study corroborates that the overall count of immune cells serves as a robust independent contributor to patient survival. When each immune cell subset was separately assessed, all were associated with an improved clinical outcome, including CD3^+^CD8^-^FoxP3^-^ and CD3^+^CD8^+^ T cells.

Chemokines and cell-cell interaction molecules play pivotal roles in tumor immunology, orchestrating the migration and interaction of immune cells within the TME, crucial for both tumor progression and anti-tumor immune responses. The role of chemokines like *CXCL10* and *CXCL11* in recruiting T cells via CXCR3 interaction is well-documented (48–50), but our data provide novel insights into their specific impact on the spatial distribution and density of T cell subsets. Specifically, the observation of a direct relationship between the expression levels of these chemokines and the proximity of T cells to tumor cells, suggesting a role in immune cell positioning. This spatial aspect is critical, as the efficacy of T cell-mediated immune responses against tumors is profoundly influenced by their infiltration and distribution patterns (51, 52). These findings support the concept that *CXCL10* and *CXCL11*, and other molecules like from the HLA family, are pivotal in shaping the immune response in the TME.

The ICR gene signature captured the presence of tumor-enriched T cell clones and outperformed conventional prognostic molecular classifications, as previously proved in breast cancer (18, 20) and our AC-ICAM consisting of 348 colon cancer patients (21). Our results support that ICR immune subtypes display different T cell densities. Additionally, the spatial positioning of immune cells relative to tumor cells is critical. This proximity can either reflect the ability of immune cells to target tumors effectively or indicate the capacity of tumor cells to modulate immune cell behavior. The spatial relationships among diverse immune cell types also shed light on their extensive interactions within the immune landscape. As immune-mediated tumor rejection becomes more pronounced (higher ICR immune subtype), it appears that effector T cells are found at greater distances from CD3^+^FoxP3^+^ T cells, which mainly perform immune regulatory function in this context. This spatial separation could potentially reduce the inhibitory effects of CD3^+^FoxP3^+^ T cells on the antitumor activities of CD3^+^CD8^-^FoxP3^-^ and CD3^+^CD8^+^ T cells, resulting in a more potent immune response against the tumor and thereby reinforcing the notion that higher ICR immune subtypes are associated with a more robust antitumor immune response (18, 20).

Long-term colon cancer survivors typically exhibit a high ICR score, characterized by an increased density of lymphocytes, including Th and CD8 T cells, which play an essential role in tumor immunity and have been associated with favorable clinical outcome in various cancer types (53–55). These T cells, according to our observation, are positioned more proximal to tumor cells in comparison to CD3^+^FoxP3^+^ T cells. Our findings highlight the importance of not only the presence but also the spatial distribution of T cell subsets within the TME, which may significantly impact the antitumor immune response and subsequent patient survival outcomes.

In conclusion, the integration of cutting-edge *in situ* analysis methodologies with advanced tissue imaging techniques offers a novel perspective for characterizing the tumor immune microenvironment in cancer, yielding more precise and clinically relevant information. This study primarily focused on major T cell subtypes, such as CD3^+^CD8^+^, CD3^+^CD8^-^FoxP3^-^, and CD3^+^FoxP3^+^ T cells, in the context of colon cancer, paving the way for future studies to assess the generalizability of these findings to other cancer types, and expand the panels to more markers to visualize other relevant immune cell types, as well as the potential role of the immune cell spatial distribution in predicting treatment response. In this study, a comprehensive analysis of clinical data, genomic profiles, and T-cell spatial distribution underscores the feasibility of integrating diverse parameters to elucidate the prognostic significance of ICR signatures, with potential applicability across other cancer types. This development is not only crucial for biomarker research, but also pivotal for comprehending the intricate biology of cancer immunity, which is instrumental in the creation of the next generation of immunotherapeutic agents.

## Data availability statement

The data presented in the study are available through controlled access at dbGaP (phs002978.v1.p1) and public access SRA (PRJNA941834), as well as via FigShare (https://doi.org/10.6084/m9.figshare.16944775).

## Ethics statement

The studies involving humans were approved by Institutional Review Board of Leiden University Medical Center Institutional Review Board of Sidra Medicine. The studies were conducted in accordance with the local legislation and institutional requirements. The participants provided their written informed consent to participate in this study.

## Author contributions

HZ: Conceptualization, Data curation, Formal analysis, Investigation, Methodology, Software, Visualization, Writing – original draft, Writing – review & editing. JR: Conceptualization, Investigation, Methodology, Project administration, Supervision, Validation, Visualization, Writing – review & editing. EA: Investigation, Methodology, Writing – review & editing. IS: Data curation, Investigation, Software, Writing – review & editing. RH: Data curation, Investigation, Software, Writing – review & editing. RV: Investigation, Supervision, Writing – review & editing. MI: Methodology, Software, Supervision, Writing – review & editing. XL: Investigation, Supervision, Writing – review & editing. Nd: Investigation, Project administration, Supervision, Writing – review & editing. RT: Conceptualization, Project administration, Resources, Supervision, Writing – review & editing. AV: Project administration, Resources, Writing – review & editing. DB: Funding acquisition, Investigation, Methodology, Project administration, Resources, Supervision, Validation, Writing – review & editing. WH: Conceptualization, Funding acquisition, Investigation, Methodology, Project administration, Resources, Supervision, Visualization, Writing – review & editing. PK: Conceptualization, Funding acquisition, Investigation, Methodology, Project administration, Resources, Supervision, Validation, Visualization, Writing – review & editing.

## Supplementary material

Supplementary Figure 1 Comparison of staining patterns between standard immunohistochemistry (IHC) and multiplex immunofluorescence (mIF). Scale bar is 100 µm.

Supplementary Figure 2 Representative images of nearest neighbor distances from T cell subsets to tumors or between different T cell subsets. **(A-G)** Nearest neighbor distances from CD3+CD8-FoxP3-, CD3^+^CD8^-^FoxP3^-^Ki67^+^, CD3^+^FoxP3^+^, CD3^+^FoxP3^+^Ki67^+^, CD3^+^CD8^+^, CD3^+^CD8^+^Ki67^+^ and CD3^+^CD8^+^GrB+ T cells to tumor cells, respectively. **(H-J)** Nearest neighbor distances from CD3^+^CD8^-^FoxP3^-^ to CD3^+^FoxP3^+^, CD3^+^CD8^+^ to CD3^+^FoxP3^+^, and CD3^+^CD8^-^FoxP3^-^ to CD3^+^CD8^+^ T cells, respectively.

Supplementary Figure 3 Heatmap representation of the correlation between ICR classification and the distances from T cell to tumor cells or the distances between different T cell subsets.

Supplementary Figure 4 Kaplan-Meier curves of overall (top-panels) and progression-free (bottom-panels) survival according to the densities of T cell subtypes **(A)** Epithelial CD3^+^CD8^-^FoxP3^-^ T cells; **(B)** Epithelial CD3^+^CD8^+^GrB^+^ T cells and the distances among different T cell subtypes or from T cells to tumor cells **(C)** CD3^+^CD8^+^ T cells to tumor; **(D)** CD3^+^CD8^-^FoxP3^-^ to CD3^+^CD8^+^ T cells. Densities and distances above the median are designated as ‘high’, while those below the median are classified as ‘low’.

Supplementary Table 1 Optimized chromogenic singleplex IHC protocols for the biomarkers in this cohort. Each singleplex (row) includes the appropriate number of antigen retrievals (AR) before and after antibody application so as to mimic a multiplex. All primary antibodies are either Mouse or Rabbit monoclonal antibodies. These were added in positions 1, 3 and 5 of the staining sequence, highlighted in green. With six biomarkers and three singeplexes per biomarker, there was a total of eighteen singleplex protocols. Fake Ab = wash buffer.

Supplementary Table 2 List of antibodies used in the study. Staining orders (first column) and dilutions of antibodies for multiplex immunofluorescence (mIF).

Supplementary Table 3 Clinicopathological and genomic characteristics of colon cancer cases. *P* values are derived from comparison between mIF and AC-ICAM cohort. AJCC, The American Joint Committee on Cancer; MSI, Microsatellite Instability; CMS, Consensus Molecular Subtypes; ICR, Immunologic Constant of Rejection; mIF, multiplex immunofluorescence; AC-ICAM, An Atlas and Compass of Immune-Cancer-Microbiome Interactions in Colon Cancer.

Supplementary Table 4 Median cell densities of T cell subsets classified according to epithelial and stromal compartments.

Supplementary Table 5 Nearest distance (median) from different T cell subsets to tumor cells and distances between T cell subsets.

Supplementary Table 6 ICR classification and correlations with clinicopathological and genomic characteristics. AJCC, The American Joint Committee on Cancer; MSI, Microsatellite Instability; CMS, Consensus Molecular Subtypes; *, One-way ANOVA test for continuous variables and Fisher’s exact test for discrete variables.

Supplementary Table 7 ICR classification and correlations with spatial distribution of T cell subsets. *, One-way ANOVA test to compare the three ICR groups; **, Student t-test to compare ICR Medium-High versus ICR Low groups.
