# Supplementary figures and images for: Location matters: spatial dynamics of tumor-infiltrating T cell subsets is prognostic in colon cancer
Source: Front Immunol. 2024 Feb 5;15:1293618. doi: 10.3389/fimmu.2024.1293618 (PMC10875018; doi:10.3389/fimmu.2024.1293618)

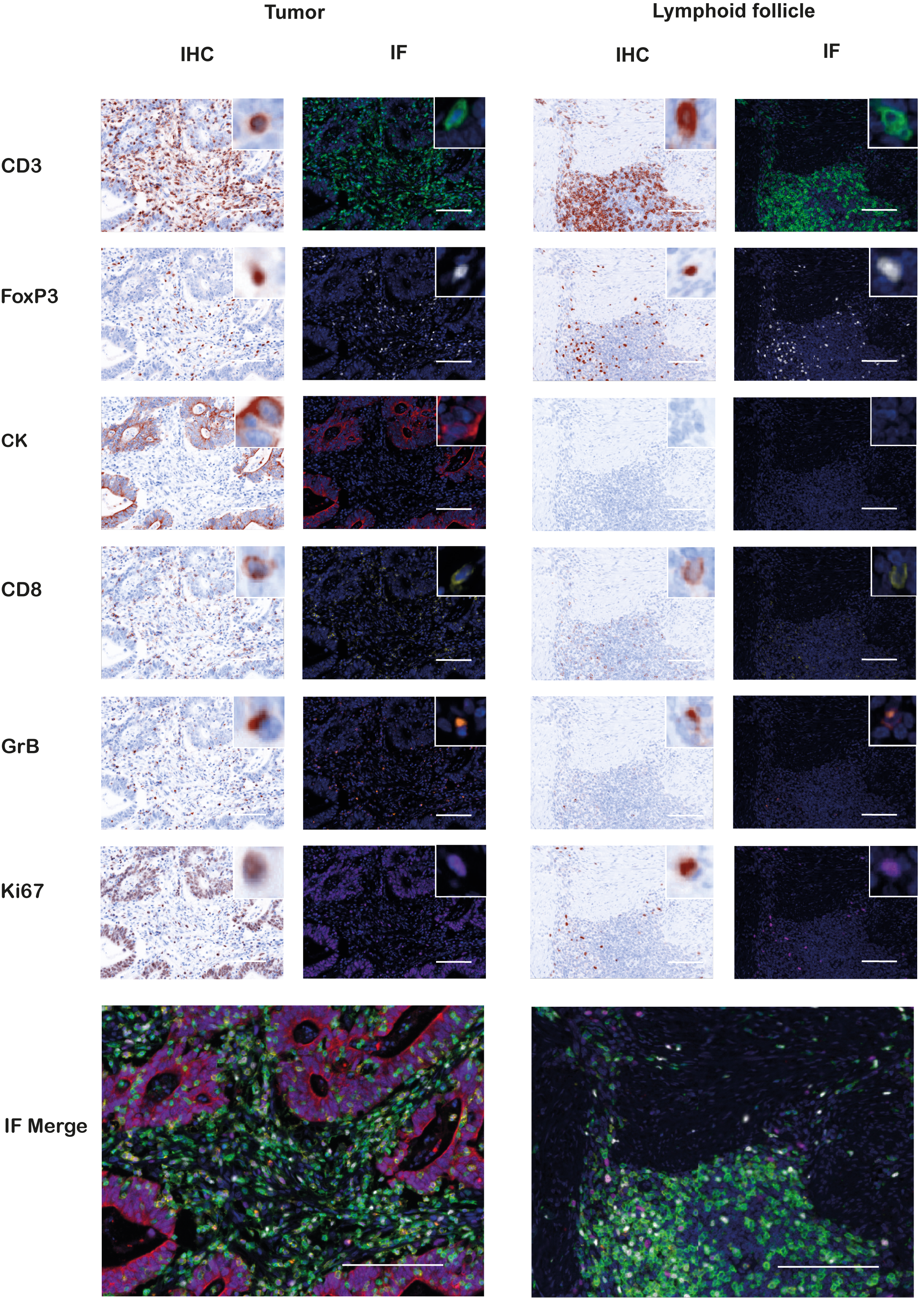

Supplement: Supplementary Figure 1 — Comparison of staining patterns between standard immunohistochemistry (IHC) and multiplex immunofluorescence (mIF). Scale bar is 100 µm. [file Image_1.tif]

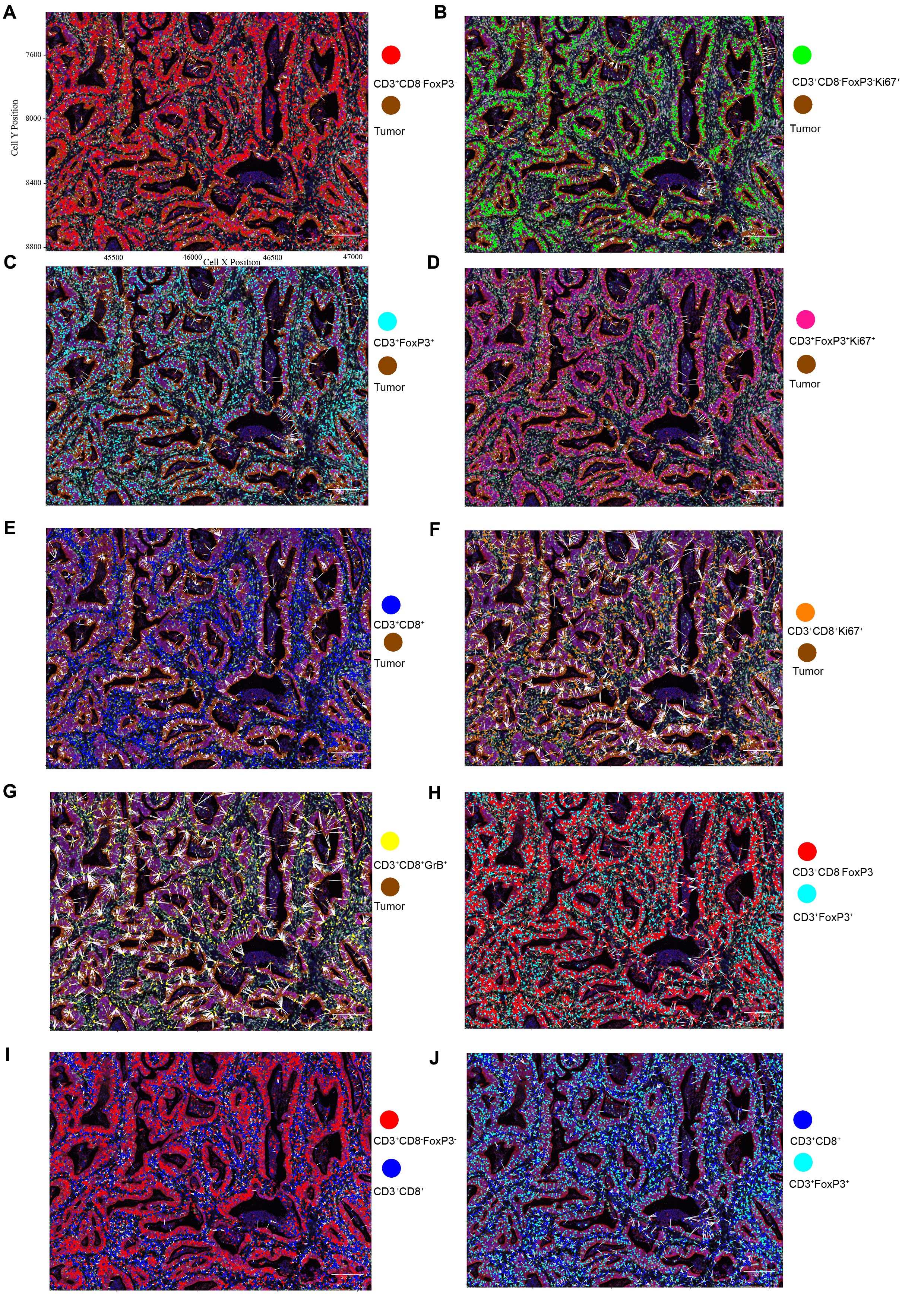

Supplement: Supplementary Figure 2 — Representative images of nearest neighbor distances from T cell subsets to tumors or between different T cell subsets. (A-G) Nearest neighbor distances from CD3+CD8-FoxP3-, CD3+CD8-FoxP3-Ki67+, CD3+FoxP3+, CD3+FoxP3+Ki67+, CD3+CD8+, CD3+CD8+Ki67+ and CD3+CD8+GrB+ T cells to tumor cells, respectively. (H-J) Nearest neighbor distances from CD3+CD8-FoxP3- to CD3+FoxP3+, CD3+CD8+ to CD3+FoxP3+, and CD3+CD8-FoxP3- to CD3+CD8+ T cells, respectively. [file Image_2.jpeg]

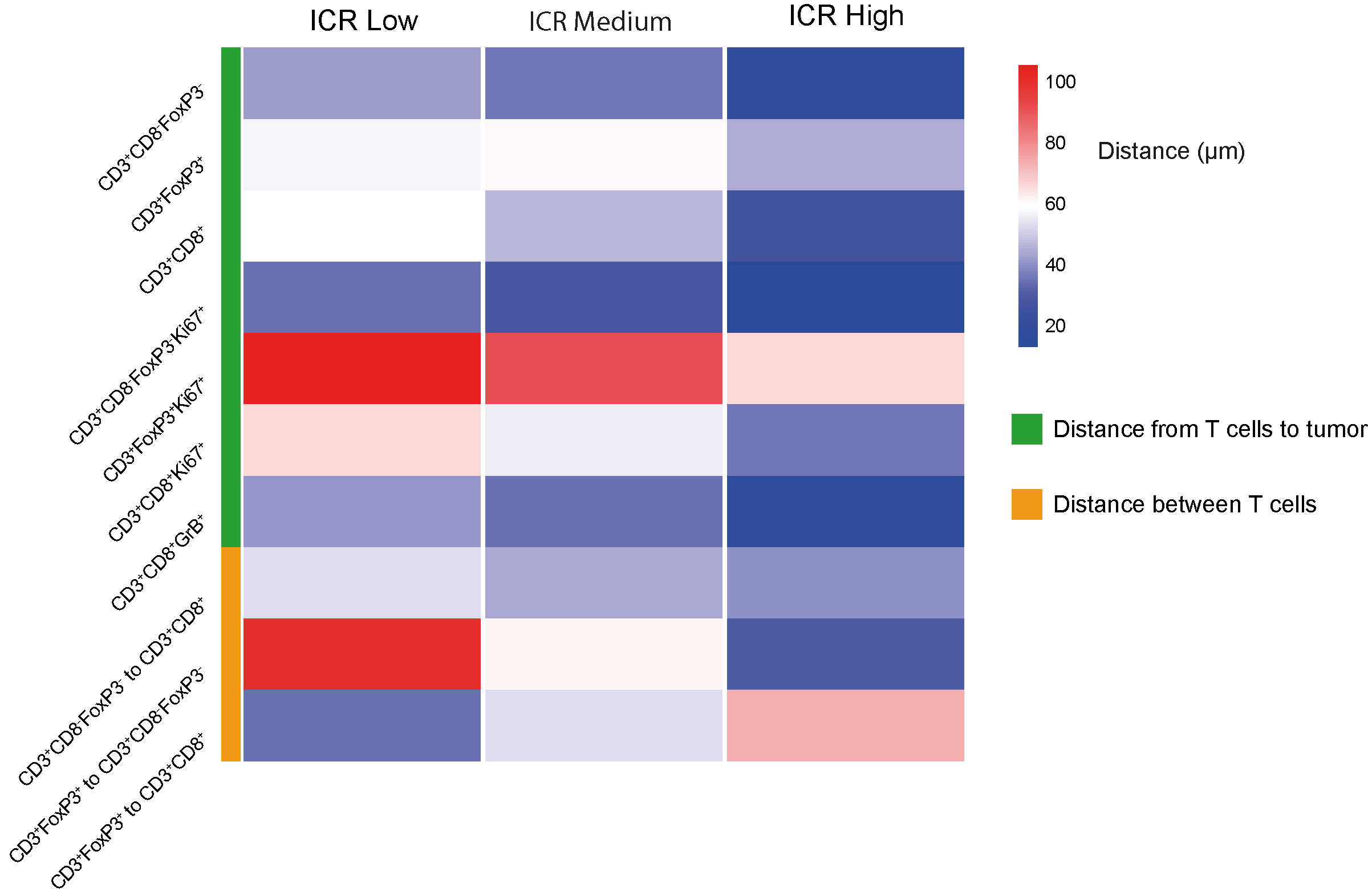

Supplement: Supplementary Figure 3 — Heatmap representation of the correlation between ICR classification and the distances from T cell to tumor cells or the distances between different T cell subsets. [file Image_3.tif]

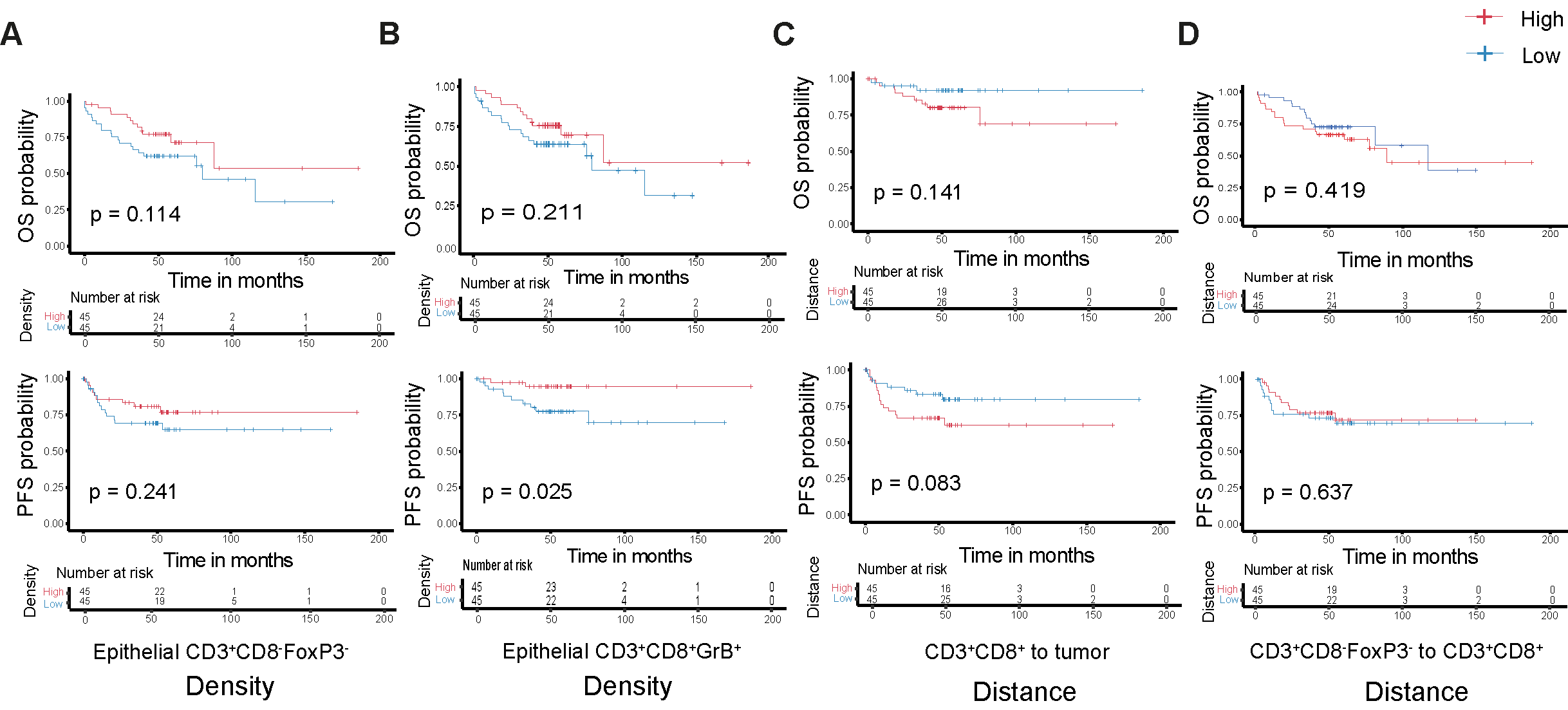

Supplement: Supplementary Figure 4 — Kaplan-Meier curves of overall (top-panels) and progression-free (bottom-panels) survival according to the densities of T cell subtypes (A) Epithelial CD3+CD8-FoxP3- T cells; (B) Epithelial CD3+CD8+GrB+ T cells and the distances among different T cell subtypes or from T cells to tumor cells (C) CD3+CD8+ T cells to tumor; (D) CD3+CD8-FoxP3- to CD3+CD8+ T cells. Densities and distances above the median are designated as ‘high’, while those below the median are classified as ‘low’. [file Image_4.tif]
