# Supplementary material for: Location matters: spatial dynamics of tumor-infiltrating T cell subsets is prognostic in colon cancer
Source: Front Immunol. 2024 Feb 5;15:1293618. doi: 10.3389/fimmu.2024.1293618 (PMC10875018; doi:10.3389/fimmu.2024.1293618)
Supplement: Supplementary Table 2 — List of antibodies used in the study. Staining orders (first column) and dilutions of antibodies for multiplex immunofluorescence (mIF). [file DataSheet_2.pdf]

Table S2

Maker	Clone	Distributor	Catlog	AR	Dilution (Ab)	Incubation time (Ab)	Opal
CD8	C8/144B	Dako	M7103	PH6	1:400	ON	620
Granzyme B (GrB)	GrB-7	Monosan	MON7029-1	PH9	1:200	1H	650
CD3	Polyclonal	Dako	A0452	PH6	1:400	ON	520
Ki-67	MIB-1	Dako	M7240	PH6	1:800	1H	690
FoxP3	236A/E7	AbCam	ab20034	PH9	1:200	ON	540
Cytokeratin (CK)	AE1/AE3	Dako	M3515	PH6	1:1600	1H	570
