# Supplementary material for: Location matters: spatial dynamics of tumor-infiltrating T cell subsets is prognostic in colon cancer
Source: Front Immunol. 2024 Feb 5;15:1293618. doi: 10.3389/fimmu.2024.1293618 (PMC10875018; doi:10.3389/fimmu.2024.1293618)
Supplement: Supplementary Table 3 — Clinicopathological and genomic characteristics of colon cancer cases. P values are derived from comparison between mIF and AC-ICAM cohort. AJCC, The American Joint Committee on Cancer; MSI, Microsatellite Instability; CMS, Consensus Molecular Subtypes; ICR, Immunologic Constant of Rejection; mIF, multiplex immunofluorescence; AC-ICAM, An Atlas and Compass of Immune-Cancer-Microbiome Interactions in Colon Cancer. [file DataSheet_3.pdf]

Characteristics	mIF cohort (90)<br>N (%)	Original AC-ICAM cohort (348)<br>N (%)	*P* value
**Median age (range), years**	69 (26-88)	68 (25-91)	0.124
**Sex**			0.278
Female	40 (44.4%)	182 (52.3%)	
Male	50 (55.6%)	166 (47.7%)	
**Anatomy location**			
Left sided	50 (55.6%)	165 (47.4%)	0.131
(flexura lienalis, colon descendens, colon sigmoideum, rectosigmoideum)			
Right sided	40 (44.4%)	183 (52.6%)	
(ceceum, colon ascendens, flexura hepatica, colon transversum)			
**Adjuvant treatment**			0.848
Yes	27 (30%)	110 (31.6%)	
No	63 (70%)	238 (68.4%)	
**AJCC staging**			0.201
I	18 (20.0%)	55 (15.8%)	
II	35 (38.9%)	122 (35.1%)	
III	26 (28.9%)	110 (31.9%)	
IV	11 (12.2%)	61 (17.2%)	
**MSI status**			0.767
MSI-H	15 (16.7%)	57 (20.3%)	
MSS	75 (83.3%)	224 (79.7%)	
**CMS classification**			0.283
CMS1	12 (13.3%)	41 (12.4%)	
CMS2	25 (27.8%)	76 (21.8%)	
CMS3	19 (21.1%)	66 (18.9%)	
CMS4	17 (18.9%)	82 (23.6%)	
Mixed	17 (18.9%)	81 (23.3%)	
**ICR classification**			0.318
Low	30 (33.3%)	86 (24.9%)	
Medium	40 (44.4%)	180 (52.0%)	
High	20 (22.2%)	80 (23.1%)	
