# Supplementary material for: Location matters: spatial dynamics of tumor-infiltrating T cell subsets is prognostic in colon cancer
Source: Front Immunol. 2024 Feb 5;15:1293618. doi: 10.3389/fimmu.2024.1293618 (PMC10875018; doi:10.3389/fimmu.2024.1293618)
Supplement: Supplementary Table 4 — Median cell densities of T cell subsets classified according to epithelial and stromal compartments. [file DataSheet_4.pdf]

Phenotype	Median cell densities by tumor compartment (cells/mm^2)	
	Epithelium	Stroma
$CD3^+$	142.4	920.1
$CD3^+Ki67^+$	81.3	209.6
$CD3^+CD8^-FoxP3^-$	89.6	595.2
$CD3^+CD8^-FoxP3^-Ki67^+$	30.2	93.3
$CD3^+FoxP3^+$	41.1	214.5
$CD3^+FoxP3^+Ki67^+$	9.1	15.0
$CD3^+CD8^+$	31.7	129.2
$CD3^+CD8^+Ki67^+$	4.7	14.3
$CD3^+CD8^+GrB^+$	13.0	32.1
