# Supplementary material for: Location matters: spatial dynamics of tumor-infiltrating T cell subsets is prognostic in colon cancer
Source: Front Immunol. 2024 Feb 5;15:1293618. doi: 10.3389/fimmu.2024.1293618 (PMC10875018; doi:10.3389/fimmu.2024.1293618)
Supplement: Supplementary Table 5 — Nearest distance (median) from different T cell subsets to tumor cells and distances between T cell subsets. [file DataSheet_5.pdf]

Nearest distance between two cells	Nearest distance in μm (median)
$CD3^+$ to tumor	53.2
$CD3^+Ki67^+$ to tumor	47.6
$CD3^+CD8^-FoxP3^-$ to tumor	34.8
$CD3^+CD8^-FoxP3^-Ki67^+$ to tumor	27.9
$CD3^+FoxP3^+$ to tumor	54.6
$CD3^+FoxP3^+Ki67^+$ to tumor	86.6
$CD3^+CD8^+$ to tumor	47.0
$CD3^+CD8^+Ki67^+$ to tumor	54.2
$CD3^+CD8^+GrB^+$ to tumor	33.9
$CD3^+CD8^-FoxP3^-$ to $CD3^+CD8^+$	56.9
$CD3^+FoxP3^+$ to $CD3^+CD8^+$	41.2
$CD3^+FoxP3^+$ to $CD3^+CD8^-FoxP3^-$	35.7
