# Supplementary material for: Location matters: spatial dynamics of tumor-infiltrating T cell subsets is prognostic in colon cancer
Source: Front Immunol. 2024 Feb 5;15:1293618. doi: 10.3389/fimmu.2024.1293618 (PMC10875018; doi:10.3389/fimmu.2024.1293618)
Supplement: Supplementary Table 6 — ICR classification and correlations with clinicopathological and genomic characteristics. AJCC, The American Joint Committee on Cancer; MSI, Microsatellite Instability; CMS, Consensus Molecular Subtypes; *, One-way ANOVA test for continuous variables and Fisher’s exact test for discrete variables. [file DataSheet_6.pdf]

Characteristics	N (%)	ICR Low N=30 (33.3%)	ICR Medium N=40 (44.4%)	ICR High N=20 (22.2%)	*P* value*
Median age (range), years	69 (26-88)	66 (39-83)	75 (53-88)	71 (26-84)	**0.040**
Sex					
Female	40 (44.4%)	8 (26.7%)	24 (60%)	8 (40%)	**0.019**
Male	50 (55.6%)	22 (73.3%)	16 (40%)	12 (60%)	
Anatomy location					
Left sided	50 (55.6%)	20 (66.7%)	19 (47.5%)	11 (55%)	0.279
Right sided	40 (44.4%)	10 (33.3%)	21 (52.5%)	9 (45%)	
Adjuvant treatment					
Yes	27 (30%)	11 (36.7%)	10 (25%)	6 (30%)	0.574
No	63 (70%)	19 (63.3%)	30 (75%)	14 (70%)	
AJCC staging					
I	18 (20.0%)	5 (16.7%)	7 (17.5%)	6 (30%)	0.460
II	35 (38.9%)	10 (33.3%)	19 (47.5%)	6 (30%)	
III	26 (28.9%)	9 (30%)	10 (25%)	7 (35%)	
IV	11 (12.2%)	6 (20%)	4 (10%)	1 (5%)	
MSI status					
MSI-H	15 (16.7%)	2 (6.7%)	6 (15%)	7 (35%)	**0.029**
MSS	75 (83.3%)	28 (93.3%)	34 (85%)	13 (65%)	
CMS classification					
CMS1	12 (13.3%)	2 (6.7%)	4 (10%)	6 (30%)	0.149
CMS2	25 (27.8%)	11 (36.7%)	12 (30%)	2 (10%)	
CMS3	19 (21.1%)	7 (23.3%)	10 (25%)	2 (10%)	
CMS4	17 (18.9%)	5 (16.7%)	6 (15%)	6 (30%)	
Mixed	17 (18.9%)	5 (16.7%)	8 (20%)	4 (20%)	
