# Supplementary material for: Location matters: spatial dynamics of tumor-infiltrating T cell subsets is prognostic in colon cancer
Source: Front Immunol. 2024 Feb 5;15:1293618. doi: 10.3389/fimmu.2024.1293618 (PMC10875018; doi:10.3389/fimmu.2024.1293618)
Supplement: Supplementary Table 7 — ICR classification and correlations with spatial distribution of T cell subsets. *, One-way ANOVA test to compare the three ICR groups; **, Student t-test to compare ICR Medium-High versus ICR Low groups. [file DataSheet_7.pdf]

Characteristics	ICR Low (N=30, 33.3%)	ICR Medium (N=40, 44.4%)	ICR High (N=20, 22.2%)	*P* value*	ICR Medium-High (N=60, 66.6%)	*P* value**
Densities of T cell subsets (range)						
$CD3^+$	89.0 (3.6-251.6)	192.5 (25.7-807.5)	610.3 (41.2-2086.0)	**0.0006**	242.7 (25.7-2086.0)	**0.0008**
$CD3^+Ki67^+$	40.8 (1.3-131.2)	112.4 (9.6-514.6)	407.5 (11.0-1586.7)	**0.0014**	170.0 (9.6-1586.7)	**0.0013**
$CD3^+CD8^-FoxP3^-$	60.6 (2.0-161.0)	119.8 (16.6-629.5)	399.9 (28.3-1161.3)	**0.0004**	152.5 (16.6-1161.3)	**<0.0001**
$CD3^+CD8^-FoxP3^-Ki67^+$	17.1 (0.4-67.5)	41.2 (4.3-239.3)	151.3 (4.5-508.4)	**0.0012**	60.8 (4.3-508.4)	**<0.0001**
$CD3^+FoxP3^+$	35.2 (0.9-120.0)	52.2 (3.6-197.7)	102.7 (19.8-253.2)	**0.0020**	53.7 (3.6-253.2)	**0.0007**
$CD3^+FoxP3^+Ki67^+$	7.2 (0.1-18.4)	15.1 (0.1-66.6)	26.1 (3.2-84.9)	**0.0025**	21.5 (0.1-84.9)	**<0.0001**
$CD3^+CD8^+$	16.5 (0.6-102.1)	55.2 (0.5-102.1)	176.1 (5.7-915.1)	**0.0132**	79.0 (0.5-915.1)	**<0.0001**
$CD3^+CD8^+Ki67^+$	4.4 (0.1-26.7)	17.9 (0.1-113.1)	67.3 (0.8-351.8)	**0.0205**	30.0 (0.1-351.8)	**0.0004**
$CD3^+CD8^+GrB^+$	14.4 (0.1-132.1)	46.8 (0.1-324.3)	173.6 (5.8-808.7)	**0.0079**	74.6 (0.1-808.7)	**0.0002**
Distances from T cell subsets to tumor						
$CD3^+CD8^-FoxP3^-$	42.3 (28.2-88.0)	36.2 (22.7-60.2)	19.1 (9.6-38.6)	**0.0269**	24.6 (9.6-60.2)	**<0.0001**
$CD3^+FoxP3^+$	57.0 (20.5-98.9)	60.9 (40.1-111.0)	45.6 (21.5-88.2)	0.7291	51.6 (21.5-111.0)	0.7589
$CD3^+CD8^+$	60.2 (36.7-114.4)	47.1 (29.5-78.3)	26.3 (16.0-38.4)	**0.0004**	33.7 (16.0-78.3)	**<0.0001**
$CD3^+CD8^-FoxP3^-Ki67^+$	35.4 (22.6-70.4)	29.0 (18.2-48.2)	13.3 (5.4-35.5)	**0.0146**	19.2 (5.4-48.2)	**<0.0001**
$CD3^+FoxP3^+Ki67^+$	105.7 (42.2-191.5)	91.4 (60.1-166.5)	66.6 (38.0-104.1)	0.0805	76.1 (38.0-166.5)	**0.0001**
$CD3^+CD8^+Ki67^+$	66.7 (44.0-137.3)	56.5 (35.4-93.9)	36.3 (21.5-137.3)	**<0.0001**	48.3 (21.5-137.3)	**<0.0001**
$CD3^+CD8^+GrB^+$	41.7 (27.5-85.8)	35.3 (22.1-58.7)	18.4 (9.1-85.8)	**0.0461**	25.7 (9.1-85.8)	**<0.0001**
Distances between T cell subsets						
$CD3^+CD8^-FoxP3^-$ to $CD3^+CD8^+$	53.3 (17.1-297.4)	44.4 (16.0-132.2)	40.1 (16.1-124.5)	0.9206	43.4 (16.0-132.2)	0.2104
$CD3^+FoxP3^+$ to $CD3^+CD8^-FoxP3^-$	30.8 (11.5-97.4)	61.8 (15.5-154.2)	100.6 (12.3-256.0)	**0.0003**	81.3 (11.5-154.2)	**0.0005**
$CD3^+FoxP3^+$ to $CD3^+CD8^+$	34.6 (16.9-102.0)	53.2 (19.2-158.7)	74.1 (16.7-149.4)	**0.0051**	66.8 (16.7-158.7)	**<0.0001**
