# Supplementary material for: Location matters: spatial dynamics of tumor-infiltrating T cell subsets is prognostic in colon cancer
Source: Front Immunol. 2024 Feb 5;15:1293618. doi: 10.3389/fimmu.2024.1293618 (PMC10875018; doi:10.3389/fimmu.2024.1293618)
Supplement: Supplementary Table 1 — Optimized chromogenic singleplex IHC protocols for the biomarkers in this cohort. Each singleplex (row) includes the appropriate number of antigen retrievals (AR) before and after antibody application so as to mimic a multiplex. All primary antibodies are either Mouse or Rabbit monoclonal antibodies. These were added in positions 1, 3 and 5 of the staining sequence, highlighted in green. With six biomarkers and three singeplexes per biomarker, there was a total of eighteen singleplex protocols. Fake Ab = wash buffer. [file DataSheet_1.pdf]

	Position 1					Position 2		Position 3		Position 4		Position 5		Position 6		
**Biomarker**	**Step 1**	**Step 2**	**Step 3**	**Step 4**	**Step 5**	**Step 6**	**Step 7**	**Step 8**	**Step 9**	**Step 10**	**Step 11**	**Step 12**	**Step 13**	**Step 14**	**Step 15**	**Step 16**
**CD3**	AR1<br>1H	Peroxide<br>block	Anti-CD3 (A0452)<br>1:200<br>O/N	Envision-HRP<br>30min	DAB	AR1<br>1H	Fake Ab<br>30min	AR1<br>1H	Fake Ab<br>30min	AR1<br>1H	Fake Ab<br>30min	AR1<br>1H	Fake Ab<br>30min	AR1<br>1H	Fake Ab<br>30min	Hematoxylin
**CD8**	AR1<br>1H	Peroxide<br>block	Anti-CD8 (M7103)<br>1:400<br>ON	Envision-HRP<br>30min	DAB	AR1<br>1H	Fake Ab<br>30min	AR1<br>1H	Fake Ab<br>30min	AR1<br>1H	Fake Ab<br>30min	AR1<br>1H	Fake Ab<br>30min	AR1<br>1H	Fake Ab<br>30min	Hematoxylin
**FoxP3**	AR2<br>1H	Peroxide<br>block	Anti-FoxP3 (ab20034)<br>1:200<br>O/N	Envision-HRP<br>30min	DAB	AR1<br>1H	Fake Ab<br>30min	AR1<br>1H	Fake Ab<br>30min	AR1<br>1H	Fake Ab<br>30min	AR1<br>1H	Fake Ab<br>30min	AR1<br>1H	Fake Ab<br>30min	Hematoxylin
**GrB**	AR2<br>1H	Peroxide<br>block	Anti-GrB (MON7029-1)<br>1:200<br>O/N	Envision-HRP<br>30min	DAB	AR1<br>1H	Fake Ab<br>30min	AR1<br>1H	Fake Ab<br>30min	AR1<br>1H	Fake Ab<br>30min	AR1<br>1H	Fake Ab<br>30min	AR1<br>1H	Fake Ab<br>30min	Hematoxylin
**Ki-67**	AR1<br>1H	Peroxide<br>block	Anti-KI67 (M7240)<br>1:1600<br>O/N	Envision-HRP<br>30min	DAB	AR1<br>1H	Fake Ab<br>30min	AR1<br>1H	Fake Ab<br>30min	AR1<br>1H	Fake Ab<br>30min	AR1<br>1H	Fake Ab<br>30min	AR1<br>1H	Fake Ab<br>30min	Hematoxylin
**CK**	AR1<br>1H	Peroxide<br>block	Anti-CK (M3515)<br>1:800<br>O/N	Envision-HRP<br>30min	DAB	AR1<br>1H	Fake Ab<br>30min	AR1<br>1H	Fake Ab<br>30min	AR1<br>1H	Fake Ab<br>30min	AR1<br>1H	Fake Ab<br>30min	AR1<br>1H	Fake Ab<br>30min	Hematoxylin

	Position 1		Position 2		Position 3					Position 4		Position 5		Position 6		
**Biomarker**	**Step 1**	**Step 2**	**Step 3**	**Step 4**	**Step 5**	**Step 6**	**Step 7**	**Step 8**	**Step 9**	**Step 10**	**Step 11**	**Step 12**	**Step 13**	**Step 14**	**Step 15**	**Step 16**
**CD3**	AR1<br>1H	Fake Ab<br>30min	AR1<br>1H	Fake Ab<br>30min	AR1<br>1H	Peroxide<br>block	Anti-CD3 (A0452)<br>1:200<br>O/N	Envision-HRP<br>30min	DAB	AR1<br>1H	Fake Ab<br>30min	AR1<br>1H	Fake Ab<br>30min	AR1<br>1H	Fake Ab<br>30min	Hematoxylin
**CD8**	AR1<br>1H	Fake Ab<br>30min	AR1<br>1H	Fake Ab<br>30min	AR1<br>1H	Peroxide<br>block	Anti-CD8 (M7103)<br>1:400<br>ON	Envision-HRP<br>30min	DAB	AR1<br>1H	Fake Ab<br>30min	AR1<br>1H	Fake Ab<br>30min	AR1<br>1H	Fake Ab<br>30min	Hematoxylin
**FoxP3**	AR1<br>1H	Fake Ab<br>30min	AR1<br>1H	Fake Ab<br>30min	AR2<br>1H	Peroxide<br>block	Anti-FoxP3 (ab20034)<br>1:200<br>O/N	Envision-HRP<br>30min	DAB	AR1<br>1H	Fake Ab<br>30min	AR1<br>1H	Fake Ab<br>30min	AR1<br>1H	Fake Ab<br>30min	Hematoxylin
**GrB**	AR1<br>1H	Fake Ab<br>30min	AR1<br>1H	Fake Ab<br>30min	AR2<br>1H	Peroxide<br>block	Anti-GrB (MON7029-1)<br>1:200<br>O/N	Envision-HRP<br>30min	DAB	AR1<br>1H	Fake Ab<br>30min	AR1<br>1H	Fake Ab<br>30min	AR1<br>1H	Fake Ab<br>30min	Hematoxylin
**Ki-67**	AR1<br>1H	Fake Ab<br>30min	AR1<br>1H	Fake Ab<br>30min	AR1<br>1H	Peroxide<br>block	Anti-KI67 (M7240)<br>1:1600<br>O/N	Envision-HRP<br>30min	DAB	AR1<br>1H	Fake Ab<br>30min	AR1<br>1H	Fake Ab<br>30min	AR1<br>1H	Fake Ab<br>30min	Hematoxylin
**CK**	AR1<br>1H	Fake Ab<br>30min	AR1<br>1H	Fake Ab<br>30min	AR1<br>1H	Peroxide<br>block	Anti-CK (M3515)<br>1:800<br>O/N	Envision-HRP<br>30min	DAB	AR1<br>1H	Fake Ab<br>30min	AR1<br>1H	Fake Ab<br>30min	AR1<br>1H	Fake Ab<br>30min	Hematoxylin

	Position 1		Position 2		Position 3		Position 4		Position 5					Position 6		
**Biomarker**	**Step 1**	**Step 2**	**Step 3**	**Step 4**	**Step 5**	**Step 6**	**Step 7**	**Step 8**	**Step 9**	**Step 10**	**Step 11**	**Step 12**	**Step 13**	**Step 14**	**Step 15**	**Step 16**
**CD3**	AR1<br>1H	Fake Ab<br>30min	AR1<br>1H	Fake Ab<br>30min	AR1<br>1H	Fake Ab<br>30min	AR1<br>1H	Fake Ab<br>30min	AR1<br>1H	Peroxide<br>block	Anti-CD3 (A0452)<br>1:200<br>O/N	Envision-HRP<br>30min	DAB	AR1<br>1H	Fake Ab<br>30min	Hematoxylin
**CD8**	AR1<br>1H	Fake Ab<br>30min	AR1<br>1H	Fake Ab<br>30min	AR1<br>1H	Fake Ab<br>30min	AR1<br>1H	Fake Ab<br>30min	AR1<br>1H	Peroxide<br>block	Anti-CD8 (M7103)<br>1:400<br>ON	Envision-HRP<br>30min	DAB	AR1<br>1H	Fake Ab<br>30min	Hematoxylin
**FoxP3**	AR1<br>1H	Fake Ab<br>30min	AR1<br>1H	Fake Ab<br>30min	AR1<br>1H	Fake Ab<br>30min	AR1<br>1H	Fake Ab<br>30min	AR2<br>1H	Peroxide<br>block	Anti-FoxP3 (ab20034)<br>1:200<br>O/N	Envision-HRP<br>30min	DAB	AR1<br>1H	Fake Ab<br>30min	Hematoxylin
**GrB**	AR1<br>1H	Fake Ab<br>30min	AR1<br>1H	Fake Ab<br>30min	AR1<br>1H	Fake Ab<br>30min	AR1<br>1H	Fake Ab<br>30min	AR2<br>1H	Peroxide<br>block	Anti-GrB (MON7029-1)<br>1:200<br>O/N	Envision-HRP<br>30min	DAB	AR1<br>1H	Fake Ab<br>30min	Hematoxylin
**Ki-67**	AR1<br>1H	Fake Ab<br>30min	AR1<br>1H	Fake Ab<br>30min	AR1<br>1H	Fake Ab<br>30min	AR1<br>1H	Fake Ab<br>30min	AR1<br>1H	Peroxide<br>block	Anti-KI67 (M7240)<br>1:1600<br>O/N	Envision-HRP<br>30min	DAB	AR1<br>1H	Fake Ab<br>30min	Hematoxylin
**CK**	AR1<br>1H	Fake Ab<br>30min	AR1<br>1H	Fake Ab<br>30min	AR1<br>1H	Fake Ab<br>30min	AR1<br>1H	Fake Ab<br>30min	AR1<br>1H	Peroxide<br>block	Anti-CK (M3515)<br>1:800<br>O/N	Envision-HRP<br>30min	DAB	AR1<br>1H	Fake Ab<br>30min	Hematoxylin
